# Mental health services for German university students: acceptance of intervention targets and preference for delivery modes

**DOI:** 10.3389/fdgth.2024.1284661

**Published:** 2024-02-14

**Authors:** Fanny Kählke, Penelope Hasking, Ann-Marie Küchler, Harald Baumeister

**Affiliations:** ^1^ TUM School of Medicine and Health, Professorship Psychology & Digital Mental Health Care, Technische Universität München, Munich, Germany; ^2^ Curtin enAble Institute, Curtin University, Perth, WA, Australia; ^3^ Department of Clinical Psychology and Psychotherapy, Ulm University, Ulm, Germany

**Keywords:** acceptance, delivery modes, intervention targets, university students, internet- and mobile-based interventions, preference, mental health care service use

## Abstract

**Introduction:**

Most university students with mental disorders remain untreated. Evaluating the acceptance of intervention targets in mental health treatment, promotion, and prevention, as well as mental health service delivery modes is crucial for reducing potential barriers, increasing healthcare utilization, and efficiently allocating resources in healthcare services.

**Aim:**

The study aimed to evaluate the acceptance of various intervention targets and delivery modes of mental health care services in German first-year university students.

**Methods:**

In total, 1,376 first-year students from two German universities from the 2017–2018 multi-center cross-sectional cohort of the StudiCare project, the German arm of the World Mental Health International College Student Survey initiative, completed a web-based survey assessing their mental health. Mental disorder status was based on self-reported data fulfilling the DSM-IV criteria. We report frequencies of accepted delivery modes [categories: group or in-person therapy with on or off campus services, self-help internet- or mobile-based intervention (IMI) with or without coaching, or a combination of a in-person and IMI (blended)]. In a multinomial logistic regression, we estimate correlates of the preference for in-person vs. IMI vs. a combination of both modes (blended) modalities. Additionally, we report frequencies of intervention targets (disorder specific: e.g., social phobia, depressive mood; study-related: test anxiety, procrastination; general well-being: sleep quality, resilience) their association with mental disorders and sex, and optimal combinations of treatment targets for each mental illness.

**Results:**

German university students' acceptance is high for in-person (71%–76%), moderate for internet- and mobile-based (45%–55%), and low for group delivery modes (31%–36%). In-person treatment (72%) was preferred over IMI (19%) and blended modalities (9%). Having a mental disorder [odds ratio (OR): 1.56], believing that digital treatments are effective (OR: 3.2), and showing no intention to use services (OR: 2.8) were associated with a preference for IMI compared to in-person modes. Students with prior treatment experience preferred in-person modes (OR: 0.46). In general, treatment targets acceptance was higher among female students and students with mental disorders. However, this was not true for targets with the highest (i.e., procrastination) and the lowest (i.e., substance-use disorder) acceptance. If only two intervention targets were offered, a combination of study-related targets (i.e., procrastination, stress, time management) would reach 85%–88% of the students.

**Conclusion:**

In-person services are preferred, yet half of the students consider using IMI, preferably aiming for a combination of at least two study-related intervention targets. Student mental health care services should offer a combination of accepted targets in different delivery modes to maximize service utilization.

## Introduction

Up to 75% of mental disorders first appear in the mid-twenties (1). In Western countries, one-third of university students have a 12-month mental disorder (2). In young adulthood, mental illness can disrupt the adoption of adult roles and identity formation (3). Alonso et al. (4) showed that about 43% of students with mental disorders report a severe role impairment related to home management, college or work issues, personal relationships, or their social life. Thus, there is a high demand for on-campus services to improve academic performance and reduce premature college drop-out (5). Yet only a fraction of those in need receive help (6, 7). Students who suffer from mental disorders and suicidal thoughts and behaviors show meager treatment rates (25%–36.3%) that increase to a maximum of 45.1% and 60% for severe cases of 12 months and lifetime disorders, respectively (8). Reasons for low treatment utilization are multifaceted, ranging from poor mental health literacy, which is fundamental for health actions (9) to stigma, limited resources on-campus, and low-risk perception (10). Reasons that prevent students from seeking help are mostly attitudinal (internal, e.g., the desire to solve problems independently) rather than structural (external, e.g., temporal or financial costs of treatment) (11, 12).

In Germany, the picture is similar: 17% of students have at least one diagnosed mental disorder (13). A report focusing on counseling services and their perception in students found that help-seeking of those in need was low and varied from 28% (depressive symptoms) to 13% (alcohol consumption). Students reported the wish to solve problems themselves (62%) or seek advice from family and friends (55%) as barriers, and they preferred in-person counseling (97%) over online-counseling (14%) (14) At universities, the Deutsches Studierendenwerk (DSW) a nationwide voluntary association offers psycho-social advice and counseling by psychologists, including the provision of further information i.e., contact of psychotherapists (15). Access to specialist care is covered by the statutory health insurance (covering 90% of the German population), yet the waiting times from initial contact to the start of psychotherapeutic treatment average 20 weeks (16). The president of the German student services released a statement that psychosocial counseling services are insufficiently prepared for the growing number of students with mental illnesses due to limited resources leading to prolonged waiting times and lack of service (17).

Internet- and mobile-based interventions (IMI) characterized by a theory base related to evidence-based psychotherapeutic models and techniques, different level of human support, various application areas and technical implementation are feasible, flexible, cost-effective, and scalable therapies that can overcome the aforementioned barriers (11, 12, 18). They are effective in treating and preventing a broad range of disorders (19, 20), yet uptake remains low as students lack knowledge and experience with IMI but show positive attitudes towards apps (i.e., future expectations and less fear of risks) (21).

An essential prerequisite to adopting and implementing interventions is the acceptance as *behavioral intention to* use them. This is the *hypothetical acceptability* referred to as willingness to use or to receive an intervention. In contrast, acceptance can also be understood or referred to as the *actual acceptability* reflecting the utilization of an intervention, as expressed in uptake rates, adherence, or reported satisfaction (22). Prior research has focused on user engagement (adherence) and acceptability of intervention utilization.

However, little is known about individuals’ acceptance (intention to use) and preferred delivery modes of mental health care services and their associated factors. Meta-analytic evidence suggests that patients receiving their preferred psycho-social treatment show less drop-out and increased therapeutic alliance (23). Research on delivery mode preference among university students is limited and shows varying results: US students preferred IMIs over in-person services (24), whereas Irish students preferred in-person services (79%) over websites for mental health (25). Examining a combination of face-to-face (F2F) and IMIs (i.e., blended delivery), Benjet et al. (26) found that Mexican university students preferred in-person and blended treatments over pure IMIs.

Understanding the characteristics of individuals who prefer certain delivery modes over others is required to further improve treatment provision. So far, only one study has examined associated factors of treatment modes in students: Benjet et al. (26) showed that depression, attention-deficit-hyperactivity disorder, beliefs about treatment efficacy, feeling embarrassed or worried about the negative consequences for one's academic career, and the desire to solve problems individually were significant predictors for students to prefer internet- or mobile-based delivery. Kozlov et al. (27) showed that the preference for digital care modalities varies in the general population and is related to symptom severity (e.g., in anxiety and depression) and demographic factors. Almost half of the participants preferred video-psychotherapy, one fourth had no preference, and all others preferred self-guided modes. Those favoring video-psychotherapy had higher symptom severity of depression and anxiety and showed a greater need for higher levels of care. Self-guided digital care was preferred by older, male participants, and those not showing depression or anxiety.

A discrete choice experiment showed that German participants prefer blended care incorporating F2F contact with a psychotherapist over other or no form of human contact in online-based programs. This preference is independent from prior treatment experience and symptom severity as well as sociodemographic parameters (28). A survey by Lincke et al. (29) likewise found a preference for human contact with most participants choosing in-person therapy (81.5%, 78.1%), some choosing blended therapy (11.2%, 11.5%) and only few choosing pure online therapy (6.7%, 7%). Low acceptance rates towards online-based treatments were not affected by the COVID-19 pandemic, but participants that were younger (14–15 years old), male or students had a higher likelihood of preferring an online therapy.

Interestingly, an internet- and mobile-based delivery mode for interventions seems to be preferred in health promotion and prevention targets as opposed to treatment targets. In one study, mobile health apps for coping with stress were preferred over medication, a psychiatrist, online self-help training, or F2F group courses, and were as likely to be used as GPs, psychologists and self-help literature (30). Taking patient preference into account when considering treatment options is important, both for treatment outcome and patient rights. Meta-analytic evidence shows that accommodating patient preferences in psychotherapy (i.e., type of medication and psychotherapy) is associated with treatment completions and positive treatment outcomes (31). Patients with depression prefer psychotherapy to medication and combined treatments options. However, these patients also like low-threshold “treatments” or behaviors, i.e., self-help books, relaxation, or talking to a friend (32).

However, little is known about individuals’ acceptance comparing intervention targets in students. A German statutory health insurance company reported on students' interest towards health-related services. Interest was highest in health promotion (mindfulness, resilience), study- and work-related targets (time and self-management), or physical activity and lowest for substance-use reduction (33). In general, female students showed higher interest than male students. This may be explained by higher barriers to help-seeking and service use for male students compared to female students: negative attitudes and low intention of help-seeking, poorer mental health literacy, conformity to traditional masculine norms, and higher public- and self-stigmatization (low self-efficacy beliefs in overcoming mental health problems) (34–36). Providing the most accepted interventions and delivery modes may help student mental health care services to increase treatment utilization, uptake, and completion.

## AIM

In this study we used an exploratory approach to examine the frequencies of the acceptance and preference towards delivery targets and formats. In a sample of German first-year students, the study's aim was to

(1) Evaluate the acceptance of intervention targets and their association with sex and mental disorder presence.
(2) Identify the best treatment combinations for students with and without a mental disorder.
(3) Determine the acceptance of and preference for different delivery modes of care on campus in association with sex and mental disorder presence.

Additionally, we explored whether potentially relevant factors such as sex, parental education, presence of a mental disorder, prior treatment experience, intention for service use, knowledge of IMIs and beliefs about their treatment efficacy were associated with treatment mode preferences (i.e., in-person vs. IMI, in-person vs. blended mode).

## Methods

### Participants and procedures

Participants from the second (2017–2018) cohort of the StudiCare project (37) received a web-based survey, via the Qualtrics survey platform as part of their participation in the World Mental Health International College Student (WMH-ICS) initiative (38). The sample was comprised of first-year students at the Friedrich-Alexander-University Erlangen-Nuremberg (FAU) and at the University of Ulm (UUlm) in Germany. All students of an undergraduate program aged ≥18 years (*n* = 9,853) were eligible for participation, which included students with previous study experience in another program. Of the 2,201 (22.3%) students starting the survey, 1,376 students (14%) completed all items. Sixty-two percent of these were female, with an overall mean age of 20.06 years (SD = 1.73). Informed consent was obtained before survey start and participation was confidential and voluntary. The research protocol was approved by the Research Ethics Committee of the FAU (12.07.2016, 193_16 B). and the UUlm (04.08.2017, 281/17).

### Measures

The web-based WMH-ICS survey (38) consisted of validated self-report measures that screened for a wide range of mental health disorders and correlates. In Germany, the survey also included items on acceptance of and preference for delivery modes of mental health services and various clinical (e.g., depression) and preventive (e.g., resilience, mindfulness) intervention targets. The different measures used in the present study are reviewed below.

#### Acceptance and preference of treatment delivery modes

Participants were introduced to different types of available treatments delivery modes with a focus on internet-based interventions, as we assumed that they knew little about them. Acceptance of and preference for seven different delivery modes of mental health services were assessed using a binary (yes vs. no) item: “*If you had an emotional problem, which mode of treatment would you like to utilize?*” Participants could indicate their preference in a drop-down list of the seven delivery modes [*group or in-person therapy with on or off campus services, self-help internet- or mobile-based intervention (IMI, i.e., digital) with or without coaching, and a combination of in-person and IMI (blended*)]. For analyses, we aggregated a categorical variable with three mutually exclusive levels: (1) in-person services (i.e., in or off campus, group therapy), (2) IMI (i.e., self-help intervention with or without coaching) and (3) blended services (in-person and IMI combined).

#### Mental health services: acceptance of intervention targets

Acceptance of various intervention targets in mental health prevention, promotion, and treatment was assessed with the following item: “For *future development of mental health services, we would like to know which of the following intervention targets you would be interested in to help you better cope with emotional and study-related problems and to promote your well-being.*” Participants were asked to indicate (i.e., yes vs. no, multiple answers) which of the following targets (treatment options) they were interested in: disorder-specific targets (reduction of social phobia, depressive mood, reduction of alcohol or cannabis consumption, body dissatisfaction, media consumption), study-related (reduction of test anxiety, procrastination, stress and time management, perfectionism reduction), or targets focusing on general well-being (improvement of sleep quality, resilience).

#### Willingness or intention to use mental health services

Participants were then asked to report their intention to use mental health services if they developed an emotional problem by answering “*If during this coming college year, you developed an emotional problem that caused you a lot of distress and interfered with your college work, how likely would you be to go to the student Counseling Center for help*?” and “*How likely would you be to go somewhere else for help, like to your doctor, a mental health professional, or a religious advisor?*”*.* This 5-point Likert scale (ranging from “Would definitely go” – “Would definitely not go”) was adapted from an assessment of risk and resilience in service members (39) and recoded into a binary variable [yes (“would definitely go”, “would likely go”) vs. no].

#### Experience and attitudes toward internet- and mobile-based interventions

In addition, knowledge of and experience with internet-and mobile-based interventions were assessed by asking the students “*Have you ever heard about internet- and mobile-based interventions? Have you ever used one?*” (yes vs. no). Additionally, students were asked to indicate their beliefs about the efficacy of IMIs by rating the following statement “*Internet-based interventions could be an effective way of improving mental health and symptoms*” on a 5-point Likert scale (“Does not apply at all” “Fully applies”). For analyses, the categories were collapsed into a binary measure [yes (“Largely applies”, “Fully applies”) vs. no].

#### Treatment utilization/experience

Mental health service utilization was assessed with the following item “*Did you ever receive psychological counseling or medication for an emotional or substance problem?*” (39). This item was shown to any student meeting criteria for any mental health disorder (over their lifetime). All persons who sought help for a mental problem (i.e., medication, counseling) in the past were coded as help-seeker (binary measure: yes vs. no).

#### Sociodemographics

Of the many variables assessed in the survey, age (continuous variable), sex (male or female), relationship status (being in a relationship, marriage vs. being single, divorced, or widowed), parental education (binary, at least one parent with college education), study type (full-time or part-time), nationality (German or other), university (FAU or UUlm), and study experience [first-time student (freshman) vs. prior university study experience] were reported to describe the sample.

#### 12-month history of or self-assessed DSM-IV diagnosis

The following 12-month DSM-IV disorders were assessed using the validated self-report Composite International Diagnostic Interview Screening (CIDI-SC) (40, 41) scales: major depressive episode (MDE), generalized anxiety disorder (GAD), panic disorder (PD), broad mania, and drug abuse or dependence (i.e., cannabis, cocaine, or any other street or prescription drug). The CIDI-SC scales conform to blinded clinical diagnoses based on the Structured Clinical Interview for DMS-IV [SCID-IV (42);] in the area under the curve (AUC) range of .70–.78 (40, 41).

Alcohol abuse or dependence was assessed using the alcohol use disorders identification test [AUDIT, (43)]. Alcohol use disorder was defined as a total score of ≥8 and a dependence score of ≥4 (44). This AUDIT version conforms with clinical diagnoses in the AUC range of 0.78–0.91 (45). Twelve-month suicidal thoughts and behaviors (STBs) were assessed using the Columbia Suicidal Severity Rating Scale [CSSRS (46),]. This modified version assessed death wish (*“Over the past 12 months, did you wish you were dead or would go to sleep and never wake up?*”), suicide plans (“*Over the past 12 months, did you think about how you might kill yourself or work out a plan of how to kill yourself?*”) and attempted suicide (“*Over the past 12 months, have you made a suicide attempt*”). The last two items (suicide plans and/or attempts) were collapsed into a binary measure (yes vs. no).

#### Any mental health problems

We created a binary variable indicating the presence of at least one mental health disorder over the past 12 months (excluding suicidal plan and attempt). All previously described DSM-IV disorders were included. Subsequently, a variable indicating the number of mental disorders present was created (3-level: one, two, three or more mental disorders).

## Statistical analysis

In total 1,376 students fully completed (i.e., no missing variables at the item level) the survey and were included for the final analyses. Specific information on the entire student population was provided by the university administrations allowing us to calculate propensity score weights to adjust for differences between the sample obtained and the entire population (47, 48). We chose predictors previously identified as relevant for mental health, such as demographic (age, sex, nationality) and study-related variables (study program, type of undergraduate degree), that were significant in predicting non-response. First, a dependent variable indicating survey (non-)response (yes = 1, no = 0) was created. Second, a binary logistic regression model was used to estimate the propensity score for each participant. Third, the model results were converted to predicted values, which were used as weights.

All analyses were conducted with the R (version 4.3.1) statistical software extended by following packages: tidyverse, mlogit, survey, gtsummary. The Holm correction was used to control for family-wise error rate and adjust for multiple testing (49). This correction is recommended as it is less conservative and more powerful than the commonly used Bonferroni correction (50). Regarding the delivery modes, we first calculated the proportion (and standard error) of those willing to use each type of mental health care service delivery mode and preference for treatment delivery mode among the total sample. Then we report the willingness to use among those with and without any of the mental disorders and females and males, testing the difference between each of these two groups with *χ*^2^ test adjusted by a design effect estimate (weights).

Second, the “dredge” function in the package MuMin in R was used to identify the best models, using the average of the best models with Δ < 2 as measured via Aikake's Information Criterion (51). We used a bivariate multinomial logistic regression to analyse the association between sex, parental education (as an indicator for socio-economic status), any 12-month mental disorder, treatment experience, treatment efficacy beliefs, and intention to use services with preference for in-person over IMIs and in-person over blended services.

Third, we calculated the proportion and standard error of the acceptance of each intervention target and calculated the willingness to use for participants with and without any 12-month mental disorder grouped by sex, testing the difference between these two variables with a *χ*^2^ test adjusted by a design effect estimate (weights).

Fourth, for each subset by sex, we tested for a difference in target acceptance between those with and without mental disorder (*χ*^2^ test adjusted by a design effect estimate). We report odds ratios as effect sizes for interpretation purposes.

Fifth, we explored the combination of treatment targets and the relative change in overall acceptance. i. e. which two interventions would lead to the highest joint acceptance. Thus, we operationalized the optimal treatment mix as the joint acceptance of at least two of the offered treatment targets. The treatment mixes with the highest acceptance are reported for all students with and without mental disorder as well as each disorder separately. A sensitivity analysis including a combination of three targets evaluated if there is a higher acceptance when offering one additional intervention.

## Results

### Sample description

The weighted and unweighted sample characteristics (*N* = 1,376), such as demographical variables, clinical variables, and experience with IMI are shown in Supplementary Tables S1, S2. Additionally, we added demographic variables on all fully eligible students, the completer and the drop-out sample in Supplementary Table S3. Students who completed the survey were on average 21.1 years old (SD 3.4), predominately German (91.20%) and showed a balanced sex ratio (female: 50.5%). Most students were freshmen (62.9%) and enrolled in a full-time program (98.7%). Forty percent of participants were in a relationship and half (48%) had at least one parent with a college degree. The prevalence of mental disorders was high with 32.3% of students meeting the clinical criteria for at least one 12-month disorder. The most prevalent disorders were MDE (20.7%), GAD (13.2%) as well as suicidal plans and attempts (10.7%). Additionally, one fourth of students (24.0%) had some prior treatment experience. In general, knowledge about IMI was low; one third (33.4%) had heard about IMI before and only three percent had used one previously.

### Willingness and preference to use treatment delivery modes

Table 1 shows the willingness to use (acceptance) and preferred treatment delivery modes among the total sample and among those with a 12-month prevalence of a mental disorder.

**Table 1:** Willingness to use (acceptance) and preferred treatment delivery modes among the total sample and among those with 12-month prevalence of a mental disorder (*N* = 1,376).

	Total, *N* = 1,376	Mental disorder *N* = 445^1^	No mental disorder *N* = 932^1^	F	*p*-value^2^	*q*-value^3^
Delivery Mode	*N*^1^ (%, SE)	*N*^1^ (%, SE)	*N*^1^ (%, SE)			
Group therapy on campus services	427 (31%; 0.01)	126 (28%; 0.02)	301 (32%; 0.03)	2.2	0.2	>0.9
Group therapy off-campus services	502 (36%; 0.01)	147 (33%; 0.02)	355 (38%; 0.02)	3.2	0.11	0.6
In-person on campus services	977 (71%; 0.01)	311 (70%; 0.02)	666 (72%; 0.02)	0.38	0.6	>0.9
In-person off-campus services	1,047 (76%; 0.01)	355 (75%; 0.02)	713 (76%; 0.02)	0.26	0.7	>0.9
Self-help internet intervention	662 (48%; 0.02)	249 (56%; 0.03)	413 (44%; 0.02)	16	<0.001	0.002
Guided internet intervention	611 (44%; 0.02)	200 (45%; 0.03)	411 (44%; 0.02)	0.07	0.8	>0.9
Blended in-person and internet intervention	781 (57%; 0.02)	245 (55%; 0.03)	536 (58%; 0.02)	0.69	0.5	>0.9
Preference	N^1^ (%^4^, SE)	N^1^ (%^4^, SE)	N^1^ (%^4^, SE)	6.7	0.074	
In-person treatment	996 (72%; 0.01)	309 (69%; 0.02)	687 (74%; 0.02)			
Digital treatment	263 (19%; 0.01)	102 (23%; 0.02)	160 (17%; 0.01)			
Blended treatment	118 (8.6%; 0.01)	34 (7.6%; 0.01)	84 (9.1%; 0.01)			

^1^ Weighted.

^2^ *χ*^2^ test adjusted by a design effect estimate.

^3^ Holm correction for multiple testing.

^4^ Percentages may not total 100 due to rounding.

Among the total sample, the highest rated delivery modes were in-person off-campus services (76%), followed by in-person on campus services (71%). In comparison, half of the students were interested in IMIs (44%–48%) and even more accepted a blended delivery mode combining in-person with digital services (57%). Group therapy on- and off-campus were the least accepted (31%–36%) modalities. In general, only very small differences in the acceptance of delivery modes were found between students with and without mental disorders. However, students with mental disorders showed lower acceptance towards group therapies (28%–33% vs. 32%–38%) and significantly higher acceptance for self-help IMI (56% vs. 44%, *p* < 0.001).

Delivery mode preferences varied: 72% indicated in-person, 19% internet-based intervention, and 8.6% blended interventions. Preferences were similar among those with and without any 12-month mental disorder (*p* > 0.05). Table 2 contains the results of a bivariate multinomial logistic regression for the relative association between preference for in-person, digital or blended mental health care services and various predictors. The final model containing the following predictor variables explained more variance compared to the baseline model (containing all candidate variables): any 12-month mental disorder, sex, intention to use services, treatment efficacy beliefs and prior treatment experience. Knowledge of and experiences with IMI and parental education did not contribute to a reduction in variance. Both digital and blended treatments were compared to in-person (reference category). A preference for digital over in-person delivery was significantly associated with efficacy beliefs of IMI, no prior treatment experience, no intention for service use, and having a mental disorder. Individuals that believe in the efficacy of IMI were significantly more likely to prefer digital over in-person delivery modes compared to individuals that thought they were ineffective (OR: 3.15, 95% 2.9, 4.35; *p* < .001). Participants with a mental disorder showed higher odds of preferring digital modalities [OR: 1.56, 95% (1.14, 2.12); *p* = .005]. Students without intentions to use services were more likely to prefer digital over in-person modes [OR: 2.75, 95% (1.95, 3.98); *p* < .001], compared to students with these intentions. Participants with treatment experience were less likely to choose digital over in-person treatment [OR: 0.48, 95% (0.31, 0.73); *p* < .001]. Preferring a blended over an in-person treatment was significantly associated with perceived IMI efficacy, indicating that individuals believing in their efficacy were significantly more likely to prefer digital over in-person services compared to individuals who thought they were ineffective [OR: 2.8, 95% (1.82, 4.3); *p* < .001]. Additional information on the model can be found in Supplementary material S4.

**Table 2:** Bivariate multinominal logistic regression of mental health care modality preferences for relative associations between preferred modes, and demographic, clinical predictors, and intention to use treatment.

Factor	Digital vs. In-person			Blended vs. In-person		
	OR^1^	95% CI^1^	*p*-value	OR^1^	95% CI^1^	*p*-value
Mental disorder						
(0) no disorder (ref)	1	–	–	1	–	–
(1) any 12-month disorder	1.556	1.140, 2.124	0.005	0.954	0.610, 1.494	0.8
Perceived efficacy of IMIs						
(0) no effect of IMI (ref)	1	–	–	1	–	–
(1) efficacious treatment	3.153	2.287, 4.347	<0.001	2.789	1.816, 4.284	<0.001
Treatment Experience						
(0) No prior treatment (ref)	1	–	–	1	–	–
(1) Prior treatment	0.464	0.306, 0.704	<0.001	0.661	0.395, 1.108	0.12
Sex						
Male (ref)	1	–	–	1	–	–
Female	0.817	0.611, 1.092	0.2	1.234	0.834, 1.826	0.3
Intention to use services						
(0) Willing to use services when needed (ref)	1	–	–	–	–	–
(1) Not willing to use services	2.749	1.949, 3.878	<0.001	1.180	0.787, 1.769	0.4

AIC = 1,976; No. Obs. = 1,376.

^1^ OR, odds ratio; CI, confidence interval; ref, reference.

### Willingness to use different treatment targets

Most students favored study-related intervention targets such as procrastination (75%), stress (73%), and time management (71%) (see Table 3). About half of the students were interested in interventions focusing on problems that commonly occur in college years, i.e., sleep (55%), social anxiety (50%), test anxiety (46%), and depression (46%). Well-being (resilience) was interesting for 54% of the students. Less prevalent behavioral problems, such as body-dissatisfaction (38%), media consumption (32%), and perfectionism (38%) received less interest. The least accepted treatment targets were cannabis use (2.4%) and alcohol use reduction (3.4%).

**Table 3:** Willingness to use (acceptance) treatment targets among those with and without 12-month mental disorder presence by sex (*N* = 1,376).

Target		Mental disorder (*N* = 445)			No mental disorder (*N* = 932)		
	Total, *N* = 1,376	Male, *N* = 221^1^	Female, *N* = 260^1^	OR^2,3^	Male, *N* = 461^1^	Female, *N* = 435^1^	OR^2,3^
	*N*^1^ (%, SE)	*N*^1^ (%, SE)	*N*^1^ (%, SE)	OR (95% CI)	*N*^1^ (%, SE)	*N*^1^ (%, SE)	OR (95% CI)
Stress	1,001 (73%; 0.01)	131 (67%; 0.04)	201 (80%; 0.03)	0.52 (0.34, 0.80)*	321 (66%; 0.03)	349 (79%; 0.02)	0.52 (0.39, 0.70)**
Stress management	981 (71%; 0.01)	129 (67%; 0.04)	187 (75%; 0.03)	0.68 (0.45, 1.03)	339 (70%; 0.03)	326 (73%; 0.02)	0.83 (0.62, 1.10)
Body-dissatisfaction	527 (38%; 0.01)	71 (36%; 0.04)	130 (52%; 0.03)	0.53 (0.36, 0.78)*	139 (28%; 0.03)	187 (42%; 0.02)	0.54 (0.41, 0.71)**
Cannabis	33 (2.4%; 0.00)	10 (5%; 0.02)	6 (2.2%; 0.01)	2.34 (0.81, 6.75)	11 (2.2%; 0.01)	7 (1.5%; 0.01)	1.46 (0.55, 3.89)
Alcohol	47 (3.4%; 0.01)	13 (6.5%; 0.02)	9 (3.5%; 0.01)	1.90 (0.79, 4.58)	18 (3.7%; 0.01)	7 (1.5%; 0.01)	2.51 (1.03, 6.11)
Procrastination	1,030 (75%; 0.01)	159 (82%; 0.04)	198 (79%; 0.03)	1.22 (0.76, 1.97)	361 (74%; 0.02)	312 (70%; 0.02)	1.20 (0.90, 1.61)
Sleep	798 (58%; 0.02)	117 (61%; 0.05)	166 (66%; 0.03)	0.79 (0.53, 1.16)	273 (56%; 0.03)	242 (55%; 0.02)	1.05 (0.81, 1.37)
Perfectionism	527 (38%; 0.01)	73 (38%; 0.04)	131 (52%; 0.03)	0.55 (0.38, 0.81)*	130 (27%; 0.03)	193 (44%; 0.02)	0.47 (0.36, 0.62)**
Social anxiety	744 (54%; 0.02)	130 (67%; 0.04)	160 (64%; 0.03)	1.17 (0.79, 1.74)	245 (50%; 0.03)	208 (47%; 0.02)	1.14 (0.88, 1.48)
Test anxiety	760 (55%; 0.02)	109 (56%; 0.04)	169 (67%; 0.03)	0.63 (0.43, 0.92)	224 (46%; 0.03)	259 (58%; 0.02)	0.60 (0.47, 0.78)**
Resilience/well-being	742 (54%; 0.02)	129 (67%; 0.04)	195 (78%; 0.03)	0.57 (0.38, 0.87)	169 (35%; 0.03)	249 (56%; 0.02)	0.41 (0.32, 0.54)**
Depression	632 (46%; 0.02)	130 (67%; 0.04)	185 (74%; 0.03)	0.74 (0.49, 1.11)	148 (30%; 0.03)	168 (38%; 0.02)	0.72 (0.54, 0.94)
Media consumption	434 (32%; 0.01)	70 (36%; 0.04)	95 (38%; 0.03)	0.93 (0.63, 1.38)	156 (32%; 0.03)	113 (25%; 0.02)	1.38 (1.04, 1.84)

OR, odds ratio; SE, standard error; CI, confidence interval.

* Applies if the post-hoc adjustment by holm is significant (*p* < 0.001).

** Applies if the post-hoc adjustment by holm is significant (*p* < 0.05).

^1^ Weighted percentages may not total 100% due to rounding.

^2^ *χ*^2^ test adjusted by a design effect estimate.

^3^ Holm correction for multiple testing.

Targets differed in their acceptance showing higher rates for students with mental disorders. The highest differences were seen for resilience (67%–78% vs. 35%–56%) and depression (67%–74% vs. 30%–38%). Also, interventions targeting procrastination (79%–82% vs.70%–74%), sleep (61%–66% vs. 55%–56%), perfectionism (38%–52% vs. 27%–44%) or social anxiety (64%–67% vs. 47%–50%) showed higher acceptance among students with mental disorder. The other targets were similarly distributed between students with compared to students without mental disorder.

Among students without a mental disorder, female students indicated significantly higher levels (*p *> 0.001) of acceptance for interventions on stress, body-dissatisfaction, perfectionism, test-anxiety, and resilience, as compared to male students. For all other targets acceptance was similar between sexes. Among students with a mental disorder, a similar pattern of acceptance could be observed. The only significantly higher level of acceptance in female students compared to male students was found for the intervention targets: stress, body-dissatisfaction, and perfectionism.

The optimal combination of intervention targets Table 4 shows preferred treatment combinations (*k* = 2, 3) among the total sample and with or without any 12-month mental disorder present. If we only had resources to offer two interventions, a combination of stress, procrastination, or time management, would reach acceptance rates of 85%–88%. Offering all three treatments would increase the joint acceptance to 91%. For students with a mental disorder a combination of procrastination and depression as treatment targets (91%), and a combination of sleep, procrastination, and resilience would lead to high acceptance rates (94%).

**Table 4:** The treatment target combinations (*k* = 2, 3) reaching the highest acceptance among the total sample and by 12-months mental disorder presence.

Two treatment targets	%	Three treatment targets	%
Total sample		Total sample	
Procrastination + Stress	88.18	Procrastination + Stress + Time management	91.49
Procrastination + Time management	86.20	Procrastination + Stress + Social anxiety	91.39
Stress + Time management	85.49	Procrastination + Stress + Sleep +	91.31
Any disorder		Any disorder	
Procrastination + Depression	90.99	Procrastination + Sleep + Resilience	94.06
Procrastination + Resilience	90.63	Procrastination + Social anxiety + Resilience	93.40
Procrastination + Social anxiety	88.83	Procrastination + Perfectionism + Depression	93.34
No disorder		No disorder	
Procrastination + Stress	87.93	Procrastination + Stress + Time management +	91.57
Procrastination + Time management	85.56	Procrastination + Stress + Test anxiety	90.85
Stress + Time management	85.84	Procrastination + Stress + Sleep	90.79

Supplementary Table S5 presents the optimal treatment mixes per 12-month mental disorder. For MDE, PD and suicide plan and/or attempt, a combination of procrastination and depression targets are most accepted (range: 92.10%–92.63%). Among participants with GAD the preferred 2-treatment mix was resilience and procrastination (94.09%) followed by stress and social anxiety. Students with drug abuse or dependence showed the highest willingness to use for interventions combining time management with stress or resilience (84.16%). Students with alcohol abuse or dependence preferred intervention targeting time management and stress (96.75%). For students with broad mania, the highest-rated 2-treatment mix was test anxiety in combination with procrastination (94.33%). A 3-treatment mix only showed a minimal increase in acceptance (range: 0%–3.24%) across all disorders.

## Discussion

### Principal findings

This study's sample showed that German university students' acceptance varies depending on the delivery mode (in-person 71%–76%, IMI 44%–48%, blended 57%, group therapy 31%–36%). Students with mental disorders indicated a higher acceptance of internet- and mobile-based self-help services compared to students with no disorder. Regarding delivery mode preference, in-person services were preferred over digital and blended modalities. High treatment efficacy beliefs, no intention for service use, having a mental disorder, and lack of treatment experience were significantly associated with preferring digital over in-person treatment.

The study is the first to evaluate the acceptance of intervention targets among students with and without a 12-month mental disorder in Germany. Study-related targets (i.e., procrastination) showed the highest acceptance, while reducing cannabis or alcohol were the least accepted targets. In general, female students with a mental disorder showed higher interest in various intervention targets. Analysis of treatment combinations, (both overall and among those without a mental disorder) indicated that a two-way combination of procrastination, stress, or time-management is favorable. Students with a mental disorder favored a combination of procrastination and depression as treatment targets. Offering three instead of two targets led to a negligible increase in acceptance.

### Comparison with prior work

Our findings are consistent with existing evidence on the acceptance of treatment modalities in the student population. One prior study involving Mexican students found similar acceptance rates for any in-person service (74%) and digital (42%) delivery mode. Likewise to our study, the acceptance for IMIs was significantly higher among students with a mental disorder (26). Moreover, in our sample in-person group therapy was the least accepted treatment mode, which may be explained by the desire to solve problem independently (11, 12).

Our findings regarding the preference for delivery modes differed, in part, from previous evidence. Students in Germany and Mexico preferred in-person treatments. However, Mexican students showed similar preference for blended (36%) and in-person therapy (38%), and low preference for IMIs (7%), and one fifth reported no preference at all (26). German adults significantly preferred blended over internet-delivered modes for programs focusing on stress coping (52), whereas German students in our sample preferred an internet-and mobile-based (19%) over a blended mode (9%). The reason for this difference remains unclear and evidence regarding the predictors of delivery mode preference is limited. We found efficacy beliefs of IMIs to be associated with blended and digital mode preference. Benjet et al. (26) also found treatment efficacy beliefs to be associated with IMI preference, while sex and parental education had no effect. Apolinário-Hagen et al. (52) showed a higher stress level to be a predictor of the preference for digital intervention modes. Similar to the findings in Apolinário-Hagen et al. (53), our study identified that having prior experience with a specific treatment mode positively predicted the preference for this mode among participants. In line with findings by Benjet et al. (26), existing hesitancy towards service use was a predictor for digital mode preference in our sample. That is, students who stated they would definitely not seek help commonly preferred to solve problems by themselves, hence preferred digital self-help intervention modes.

Our findings are supported by data on the general German population which prefers in-person therapy. Lincke et al. (29) found younger age and student status to be positively associated with IMI preference which may explain why we found blended therapy to be the least preferred option. However, a discrete choice experiment showed blended therapy to be the preferred mode. Unfortunately, the study only measured preferences towards IMIs and did not include in-person therapy as a selectable option (28).

Common reasons for the low acceptance and preference of IMIs are low efficacy beliefs, confidentiality and privacy concerns, scepticism about self-guided IMIs and low motivation (54). This is underscored by a relatively low uptake of reimbursable digital health applications (DiGA) that were introduced via the Digital Healhcare Act (Digitale-Versorgungs-Gesetz; DVG) in 2019. The monthly number of used DiGAs tripled from 5.000 in December 2021 to 15.000 in September 2023 (55). However, a statutory health insurance remarked that the uptake of DiGAs is relatively low and growth may be due to increased uptake of existing interventions (56).

Our study was the first to evaluate the acceptance of different treatment targets and complements previous findings on student's interest in health services and research on patient preferences for psychotherapy treatment options. In the present study acceptance was highest for targets related to problems at campus (study-related e.g., stress, procrastination) especially among students with a mental disorder, and lowest for alcohol and cannabis reduction. Procrastination, which is very prevalent in student populations and associated with lower quality of life, symptoms of stress, depression, and anxiety in university students (56), was of particular interest for students. Students with a mental disorder showed a higher acceptance of treatment targets than student without any mental disorder. This aligns with findings from two prior studies: first, Canadian students with high self-reported symptoms use more health care services (60). Second, a recent report on student health by a German statutory health insurance provider (TK), (33) showed high interest in resilience, mindfulness, and time and self-management, and low interest in substance abuse, and more interest among female students than male students. Acceptance rates of other targets, such as sleep, and media consumption, were similar to our results. In the same report, students identified study-related problems as the causes of stress they are exposed to at university. Research shows that primary care patients favor treatments that help them understand the causes of their feelings and problems and that they like to learn new skills and relaxation techniques (58). Following this logic, procrastination, stress and time-management interventions may constitute the favored treatments for university students as they help them to decrease their main stressors and increase their coping skills.

### Limitations

Some limitations of the present study should be discussed. First, the intention to use a treatment target or delivery mode does not fully translate to actual utilization behavior, i.e., the intention-behavior gap (60). We were unable to assess differences between willingness and preference to use treatment targets compared to the actual treatment utilization data. This was due to the cross-sectional design and limited access to service use data. Future studies with a longitudinal design and access to actual treatment utilization may address this issue. Yet, intention can be used as proxy for treatment utilization. Past research shows that most students expressing an interest in a digital stress management intervention also registered for it (61). Second, the drop-out was substantial due to the length of the survey as part of the WMH-ICS project and the exclusion of incomplete surveys. Although we attempted to address this issue by applying propensity score weighing, less drop-out may have revealed additional insights. Third, participants had no response category for treatment rejection, nor did we directly ask about the preferred treatment target. This may have led to skewed results, forcing students to select a response that did not reflect their true preference. Fourth, not all predictors that could be relevant for the acceptance and preference of delivery modes were included in the survey. Adding new predictors, such as fear of stigma and preference to handle problems on one's own, might help to further investigate delivery mode preferences and improve treatment provision. Fifth, due to the high drop-out and potential occurrence of selection bias, the generalizability is limited. This is especially true as both universities are in the south of Germany and results may therefore not be generalizable to all German universities. Sixth, the use of accepted and preferred intervention targets and delivery modes is a good way to increase the mental health service use. However, we do not know if these interventions are also the most effective treatments. Therefore, clinical trials need to confirm whether there is a difference in the effectiveness of preferred treatments compared to treatments that are recommended by an expert or clinician. Seventh, due to the relatively old data, preferences and attitudes may have changed over time, as efforts for the digitalization of the health care sector have sharply increased, for example, due to the COVID-19 pandemic. However, a more recent qualitative study on digital mental health services showed that German students only have little experience with this kind of services (62). Eighth, the 12-month mental disorders were solely based on self-reported DSM-IV criteria which may have led to inflated prevalences due to self-selection and recall bias. Despite these limitations, this study provides new insights and evidence regarding the acceptance for treatment targets and their delivery modes.

### Clinical implications and future research

Based on our findings, recommendations for future research and clinical implications can be made. Offering students with mental disorders their preferred treatment option minimizes their treatment reluctancy and increases their intention to use treatment while reducing mental health symptoms. Meta-analytic evidence on in-person or internet-based interventions targeting resilience and stress management supports their efficacy in reducing symptoms of depression, anxiety, and stress (63, 64). This concept is known as indirect prevention and treatment which focuses on intervention targets that are less stigmatized, but still related to disorders. In depression such intervention targets are insomnia and stress (65). Thus, future research should include more prevention and health promoting targets to assess their acceptance and preference relative to the others.

Acceptance of IMIs is moderate among students, but their preference is much lower than for in-person treatments. Given the limited resources of student counseling centers and the growing number of students with mental health problems, IMIs could still be useful. IMIs are similarly effective as face-to-face counseling in the treatment of mental disorders (69) and mental health promotion (64) and prevention (66); they are low-threshold, can reach students (with mental disorders, no intention of seeking help, and no treatment experience) who would otherwise not receive care, and have the potential to be cost-effective (68). Meta-analytic evidence of IMIs in routine care shows promising effects in the treatment of mental disorders in adults (69). However, the reach (initial contact with service) among university students remains low while the uptake of those enrolled in the interventions is high (70). Stakeholders view data security, privacy concerns and limited in-person contact as barriers for a successful implementation (71). Facilitators of implementation are evidence-based, attractive and updated IMIs adapted for contextual factors (71) and the use of evidence-based frameworks such as RE-AIM (reach, effectiveness, adoption, implementation, and maintenance) (72) or the Consolidated Framework for Implementation Research (73). IMIs, as scalable, low-threshold interventions that can be easily tailored, are an indispensable ingredient of sustainable student health care management assuming their successful implementation.

Research shows that students who cannot be offered immediate in-person treatment would prefer digital treatment to waiting (74). Students are not inherently averse to using IMI, but limited knowledge about and experiences with IMI demonstrated in our study and many others may explain students' preferences for in-person delivery modes as “the” standard treatment.

Another barrier for IMI preference could be scepticism towards and perceived risk of use, as shown in mental health apps (75). The same authors who validated the Unified Theory of Acceptance and Use of Technology (UTAUT) model for digital health care also found internet/technology anxiety (“fear or mistrust experienced while using the internet”) to be a moderating factor for the acceptance of IMIs (76). Concerns about the protection of sensitive data in digital health apps are well-known (77) and relevant to students. This is supported in a study by Dederichs et al. (78) where students emphasized data security and the scientific evaluation of IMIs as relevant topics for mHealth app development.

One strategy to increase participants’ willingness to use, treatment uptake, and treatment adherence of IMIs are acceptance facilitating interventions (AFI). We suggest AFIs that focus on providing knowledge on the effectiveness of IMIs, intervention procedures, and data security to reduce the fear of technology. This may help to strengthen positive attitudes (e.g., awareness, treatment efficacy beliefs) toward IMIs, which are known to be strong predictors for their use (52). There is evidence that AFIs can increase the acceptance of IMIs. Ebert et al. (11, 12) found internet-based personalized feedback on symptom severity and information on available services, which was integrated in our survey, to be effective in increasing help-seeking intentions. In another survey, students received different informational materials on a digital resilience training. Here, the intention to use services was associated with a higher level of stress and self-identification with testimonials. Therefore, information must be adapted to the student setting. Interestingly, most students who were offered an intervention shortly after exposure to an AFI signed up for it (61). Another promising method to increase the acceptance of IMIs involves participatory research design. Dederichs et al. (78) conducted co-design workshops to identify medical students' preferences and ideas for mobile health apps to increase their acceptance, demonstrating the feasibility and acceptance of such workshops.

In conclusion, students are interested in different intervention targets and delivery modes, partly depending on mental disorder status, treatment experience, sex, and their knowledge of treatment options. Offering one-size-fits-all interventions which are currently widely implemented in student mental health care do not match our findings. Thus, we recommend further research on the preference and acceptance of treatment targets and delivery modes in form of needs assessments to confirm and extend the available evidence. In practice, we recommend decision-makers and practitioners to follow five steps to increase the acceptance of mental health care services at university: first, evaluate current services used. Second, decide if services should cater to all students or specific target groups only (e.g., students with mental disorders). Third, choose the most accepted interventions and delivery modes. Fourth, plan and conduct AFI in general (i.e., on mental health literacy, efficacy of treatments) or specifically for delivery modes that are available and scalable, but not the most accepted (e.g., on efficacy beliefs and data protection of IMI). Fifth, if AFI are used, available services should be offered directly afterwards.

Designing and building needs-based student mental health care services, while respecting different student groups’ diverse acceptance of and preference towards treatment targets and delivery modes improves the provision of optimal treatments. This increases engagement and service use, reduces treatment reluctance, improves mental health, avoids premature college drop-out, and allocates limited resources in the best possible way.

## Data Availability

The original contributions presented in the study are included in the article/Supplementary material, further inquiries can be directed to the corresponding author.
