# Supplementary material for: Mental health services for German university students: acceptance of intervention targets and preference for delivery modes
Source: Front Digit Health. 2024 Feb 14;6:1284661. doi: 10.3389/fdgth.2024.1284661 (PMC10903098; doi:10.3389/fdgth.2024.1284661)
Supplement: Supplementary file 1 [file Table1.docx]

### Supplementary Material

**Table S1** Weighted sociodemographic, college-related variables and clinical characteristics (N = 1,376)

| **Variables** | | **N (%)** |
| --- | --- | --- |
| **Demographic variables** |  |  |
| Age (years) | M (SD), Range (years) | 21.1 (3.4), (18-36) |
| Sex | Female | 695 (50.5) |
|  | Male | 681 (49.5) |
| Study experience | Freshmen | 865 (62.9) |
|  | Higher semester | 511 (36.1) |
| University | FAU | 1,120 (81.4) |
|  | Ulm | 256 (18.6) |
| Study type | Full-time | 1,358 (98.7) |
|  | Part-time | 18 (1.3) |
| Being in relationship | Yes | 548 (39.8) |
| Parental college education | Yes | 674 (48.9) |
| Nationality | German | 1,265 (91.9) |
|  | Other | 111 (8.1) |
| **Clinical variables** |  |  |
| Treatment experience | Yes | 331 (24.0) |
| Any 12-month disorder | Yes | 481 (32.3) |
| Generalized anxiety disorder^a^ | Yes | 182 (13.2) |
| Major depressive disorder^a^ | Yes | 285 (20.7) |
| Panic disorder^a^ | Yes | 90 (6.5) |
| Broad Mania^a^ | Yes | 42 (3.1) |
| Alcohol dependency^a^ | Yes | 39 (2.8) |
| Drug dependency^a^ | Yes | 42 (3.1) |
| Suicide plans and/or attempts^a^ | Yes | 147 (10.7) |
| **Experience with IMI** |  |  |
| Have you ever heard about IMI? | Yes | 459 (33.4) |
|  | No | 917 (66.6) |
| Have you ever used an IMI? | Yes | 38 (2.7) |
|  | No | 1,339 (97.3) |

^a^ 12-month prevalence

M, Mean; SD, Standard Deviation; GAD, generalized anxiety disorder; MDE, Major Depressive Episode; FAU, Friedrich-Alexander-Universität Erlangen-Nürnberg, Ulm, Universität Ulm, IMI, internet- and mobile-based intervention

**Table S2** Unweighted sociodemographic, college-related variables and clinical characteristics (N = 1,376)

| **Variables** | | **N (%)** |
| --- | --- | --- |
| **Demographic variables** |  |  |
| Age (years) | M (SD), Range (years) | 20.62 (3.12), (18-36) |
| Sex | Female | 858 (62.4) |
|  | Male | 518 (37.6) |
| Study experience | Freshmen | 1,037 (75.4) |
|  | Higher semester | 339 (24.6) |
| University | FAU | 963 (70.0) |
|  | Ulm | 413 (30.0) |
| Study type | Full-time | 1,357 (98.6) |
|  | Part-time | 19 (1.4) |
| Being in relationship | Yes | 550 (40.0) |
| Parental college education | Yes | 674 (48.0) |
| Nationality | German | 1265 (91.9) |
|  | Other | 111 (8.1) |
| **Clinical variables** |  |  |
| Treatment experience | Yes | 332 (24.1) |
| Any 12-month disorder | Yes | 440 (32.0) |
| Generalized anxiety disorder^a^ | Yes | 189 (13.7) |
| Major depressive disorder^a^ | Yes | 298 (21.7) |
| Panic disorder^a^ | Yes | 90 (7) |
| Broad Mania^a^ | Yes | 42 (3) |
| Alcohol dependency^a^ | Yes | 39 (3) |
| Drug dependency^a^ | Yes | 42 (3) |
| Suicide plans and/or attempts^a^ | Yes | 147 (11) |
| **Experience with IMI** |  |  |
| Have you ever heard about IMI? | Yes | 471 (34.2) |
|  | No | 905 (65.8) |
| Have you ever used an IMI? | Yes | 35 (2.5) |
|  | No | 1,341 (97.5) |

^a^ 12-month prevalence

M, Mean; SD, Standard Deviation; GAD, generalized anxiety disorder; MDE, Major Depressive Episode; FAU, Friedrich-Alexander-Universität Erlangen-Nürnberg, Ulm, Universität Ulm, IMI, internet- and mobile-based intervention

**Table S3** Descriptive data on age, sex, field of program, degree, freshman status, nationality, attended university by full eligible, completer and dropout sample.

| **Variable** | | **Completer** | | **Drop-Out** | | **Full Sample** | |
| --- | --- | --- | --- | --- | --- | --- | --- |
|  |  | N | % | N | % | N | % |
|  |  | **1376** |  | **8477** |  | **9853** | **100%** |
| **Field of study** |  |  |  |  |  |  |  |
|  | Social Science | 184 | 13,4 | 890 | 10,5 | 1074 | 10,9 |
|  | Engineering | 135 | 9,8 | 1331 | 15,7 | 1466 | 14,9 |
|  | Culture & Art | 23 | 1,7 | 169 | 2,0 | 192 | 1,9 |
|  | Medicine & Health | 162 | 11,8 | 569 | 6,7 | 731 | 7,4 |
|  | Science | 295 | 21,4 | 1345 | 15,9 | 1640 | 16,6 |
|  | Economics | 151 | 11,0 | 1009 | 11,9 | 1160 | 11,8 |
|  | Languages | 86 | 6,3 | 534 | 6,3 | 620 | 6,3 |
|  | Law | 57 | 4,1 | 674 | 8,0 | 731 | 7,4 |
|  | Informatics | 123 | 8,9 | 858 | 10,1 | 981 | 10,0 |
|  | Teacher | 160 | 11,6 | 1098 | 13,0 | 1258 | 12,8 |
| **Degree** |  |  |  |  |  |  |  |
|  | BA Arts | 326 | 23,7 | 2325 | 27,4 | 2651 | 26,9 |
|  | BA Science | 672 | 48,8 | 3801 | 44,8 | 4473 | 45,4 |
|  | BA Education | 161 | 11,7 | 1107 | 13,1 | 1268 | 12,9 |
|  | BA of Law | 217 | 15,8 | 1244 | 14,7 | 1461 | 14,8 |
| **Age** |  |  |  |  |  |  |  |
|  | 17-18 | 254 | 18,5 | 1177 | 13,9 | 1431 | 14,5 |
|  | 19-20 | 416 | 30,2 | 2129 | 25,1 | 2545 | 25,8 |
|  | 20 | 257 | 18,7 | 1546 | 18,2 | 1803 | 18,3 |
|  | 21 | 124 | 9,0 | 841 | 9,9 | 965 | 9,8 |
|  | 22-23 | 130 | 9,4 | 1003 | 11,8 | 1133 | 11,5 |
|  | 24-26 | 109 | 7,9 | 966 | 11,4 | 1075 | 10,9 |
|  | 27-75 | 86 | 6,1 | 812 | 9,6 | 896 | 9,1 |
| **Sex** |  |  |  |  |  |  |  |
|  | Male | 518 | 37,6 | 4501 | 53,1 | 5019 | 50,9 |
|  | female | 858 | 62,4 | 3976 | 46,9 | 4834 | 49,1 |
| **Nationality** |  |  |  |  |  |  |  |
|  | German | 1265 | 91,9 | 7723 | 91,1 | 8988 | 91,2 |
|  | Other nationality | 111 | 8,1 | 754 | 8,9 | 865 | 8,8 |
| **University (freshman)** |  |  |  |  |  |  |  |
|  | FAU freshman | 684 | 49,7 | 4075 | 48,1 | 4759 | 48,3 |
|  | UUlm freshman | 353 | 25,7 | 1034 | 12,2 | 1387 | 14,1 |
|  | UUlm 2^nd^ year or higher | 60 | 4,4 | 296 | 3,5 | 356 | 3,6 |
|  | FAU 2^nd^ year or higher | 279 | 20,3 | 3072 | 36,2 | 3351 | 34,0 |

UUlm, University of Ulm; FAU, Friedrich-Alexander-Universität Erlangen-Nürnberg, BA, Bachelor

Table S5 The two optimal treatment mixes per various mental health disorder

| **Target** | **%** |  |  | **Target** | **%** | | |
| --- | --- | --- | --- | --- | --- | --- | --- |
| GAD |  |  |  | MDE |  | | |
| Resilience + Procrastination | 94.09 |  |  | Depression + Procrastination | 92.10 | | |
| Stress + Social anxiety | 93.14 |  |  | Procrastination + Resilience | 91.41 | | |
|  |  |  |  |  |  |  |  |
| Alcohol abuse or dependence |  |  |  | PD |  |  |  |
| Stress + Time management | 96.75 |  |  | Procrastination + Depression | 92.38 | | |
| Media consumption + Time management | 94.69 |  |  | Procrastination + Social anxiety | 92.04 | | |
|  |  |  |  |  |  | | |
| Broad Mania |  |  |  | Suicide plan and / or attempt |  | | |
| Test anxiety + Procrastination | 94.33 |  |  | Procrastination + Depression | 92.63 | | |
| Body dissatisfaction + Procrastination | 91.69 |  |  | Procrastination + Social anxiety | 91.60 | | |
|  |  |  |  |  |  | | |
| Drug abuse or dependence |  |  |  |  |  | | |
| Time management + Resilience | 84.16 |  |  |  |  | | |
| Time management + Stress | 84.16 |  |  |  |  | | |
|  |  |  |  |  |  | | |

GAD, generalized anxiety disorder; MDE, Major Depressive Episode, PD, panic disorder
