# Supplementary material for: Mental health services for German university students: acceptance of intervention targets and preference for delivery modes
Source: Front Digit Health. 2024 Feb 14;6:1284661. doi: 10.3389/fdgth.2024.1284661 (PMC10903098; doi:10.3389/fdgth.2024.1284661)
Supplement: Supplementary file 2 [file Datasheet1.pdf]

Multinominal analysis

Fanny Kählke

Find the best multilog model!

```
print(model_dredge)
```

```
## Global model call: multinom(formula = preference_modes_collapsed ~ (treatment_experience
 +
##     intention + sex + parenteducation + Any_12M_dx +
efficacy_internet_intervention +      IMI_known), data = acceptance_data,
##
weights = weight,
##
na.action = na.fail)
## Model selection table
##(Int) Any_12M_dx eff_intrn_intrv sex IMI_known prn trt_exp int df           logLik    AI
C  delta weight
## 99      +          +               +                            +   + 10  -977.703 1975.
4   0.00  0.363
## 103     +          +               +   +                        +   + 12  -975.913 1975.
8   0.42  0.294
## 107     +          +               +                +           +   + 12  -977.462 1978.
9   3.52  0.063
## 98      +                          +                            +   +  8  -981.612 1979.
2   3.82  0.054
## 111     +          +               +   +            +           +   + 14  -975.670 1979.
3   3.93  0.051
## 115     +          +               +                    +       +   + 12  -977.695 1979.
4   3.98  0.050
## 119     +          +               +   +                +       +   + 14  -975.902 1979.
8   4.40  0.040
## 102     +                          +   +                        +   + 10  -980.008 1980.
0   4.61  0.036
## 106     +                          +                +           +   + 10  -981.397 1982.
8   7.39  0.009
## 123     +          +               +                +   +       +   + 14  -977.457 1982.
9   7.51  0.009
## 114     +                          +                    +       +   + 10  -981.604 1983.
2   7.80  0.007
## 127     +          +               +   +            +   +       +   + 16  -975.664 1983.
3   7.92  0.007
## 110     +                          +   +            +           +   + 12  -979.792 1983.
6   8.18  0.006
## 118     +                          +   +                +       +   + 12  -979.998 1984.
0   8.59  0.005
## 122     +                          +                +   +       +   + 12  -981.393 1986.
8  11.38  0.001
## 71      +          +               +   +                            + 10  -983.602 1987.
2  11.80  0.001
## 126     +                          +   +            +   +       +   + 14  -979.787 1987.
6  12.17  0.001
## 70      +                          +   +                            +  8  -985.848 1987.
```

```
7  12.29  0.001
## 66      +                          +                                +  6  -987.961 1987.
9  12.52  0.001
## 67      +          +               +                                +  8  -986.077 1988.
2  12.75  0.001
## 79      +          +               +   +            +               + 12  -983.318 1990.
6  15.23  0.000
## 78      +                          +   +            +               + 10  -985.571 1991.
1  15.74  0.000
## 87      +          +               +   +                +           + 12  -983.595 1991.
2  15.78  0.000
## 74      +                          +                +               +  8  -987.691 1991.
4  15.97  0.000
## 75      +          +               +                +               + 10  -985.801 1991.
6  16.20  0.000
## 86      +                          +   +                +           + 10  -985.843 1991.
7  16.28  0.000
## 82      +                          +                    +           +  8  -987.957 1991.
9  16.51  0.000
## 83      +          +               +                    +           + 10  -986.071 1992.
1  16.74  0.000
## 95      +          +               +   +            +   +           + 14  -983.314 1994.
6  19.22  0.000
## 94      +                          +   +            +   +           + 12  -985.569 1995.
1  19.73  0.000
## 90      +                          +                +   +           + 10  -987.688 1995.
4  19.97  0.000
## 91      +          +               +                +   +           + 12  -985.798 1995.
6  20.19  0.000
## 35      +          +               +                            +      8  -996.219 2008.
4  33.03  0.000
## 39      +          +               +   +                        +     10  -994.344 2008.
7  33.28  0.000
## 43      +          +               +                +           +     10  -995.958 2011.
9  36.51  0.000
## 47      +          +               +   +            +           +     12  -994.080 2012.
2  36.75  0.000
## 51      +          +               +                    +       +     10  -996.196 2012.
4  36.99  0.000
## 55      +          +               +   +                +       +     12  -994.320 2012.
6  37.23  0.000
## 59      +          +               +                +   +       +     12  -995.940 2015.
9  40.47  0.000
## 63      +          +               +   +            +   +       +     14  -994.061 2016.
1  40.71  0.000
## 34      +                          +                            +      6 -1003.302 2018.
6  43.20  0.000
## 38      +                          +   +                        +      8 -1001.708 2019.
4  44.01  0.000
## 42      +                          +                +           +      8 -1003.050 2022.
1  46.69  0.000
## 50      +                          +                    +       +      8 -1003.277 2022.
6  47.15  0.000
## 46      +                          +   +            +           +     10 -1001.455 2022.
9  47.50  0.000
## 54      +                          +   +                +       +     10 -1001.682 2023.
4  47.96  0.000
## 58      +                          +                +   +       +     10 -1003.031 2026.
```

```
1  50.65  0.000
## 62      +                          +   +            +   +       +     12 -1001.435 2026.
9  51.46  0.000
## 101     +          +                   +                        +   + 10 -1004.829 2029.
7  54.25  0.000
## 97      +          +                                            +   +  8 -1007.048 2030.
1  54.69  0.000
## 109     +          +                   +            +           +   + 12 -1004.579 2033.
2  57.75  0.000
## 117     +          +                   +                +      +   + 12 -1004.763 2033.
5  58.12  0.000
## 105     +          +                                +           +   + 10 -1006.770 2033.
5  58.13  0.000
## 113     +          +                                    +      +   + 10 -1006.991 2034.
0  58.57  0.000
## 7       +          +               +   +                              8 -1009.082 2034.
2  58.76  0.000
## 96      +                                                      +   +  6 -1011.843 2035.
7  60.28  0.000
## 100     +                              +                       +   +  8 -1009.850 2035.
7  60.29  0.000
## 3       +          +               +                                  6 -1012.158 2036.
3  60.91  0.000
## 125     +          +                   +            +   +      +   + 14 -1004.509 2037.
0  61.61  0.000
## 6       +                          +   +                              6 -1012.586 2037.
2  61.76  0.000
## 15      +          +               +   +            +                10 -1008.692 2037.
4  61.98  0.000
## 121     +          +                                +   +      +   + 12 -1006.711 2037.
4  62.02  0.000
## 23      +          +               +   +                +            10 -1009.075 2038.
2  62.74  0.000
## 2       +                          +                                  4 -1015.118 2038.
2  62.83  0.000
## 104     +                                           +           +   +  8 -1011.604 2039.
2  63.80  0.000
## 108     +                              +            +           +   + 10 -1009.634 2039.
3  63.86  0.000
## 11      +          +               +                +                 8 -1011.774 2039.
5  64.14  0.000
## 116     +                              +                +      +   + 10 -1009.789 2039.
6  64.17  0.000
## 112     +                                               +      +   +  8 -1011.790 2039.
6  64.17  0.000
## 19      +          +               +                    +             8 -1012.154 2040.
3  64.90  0.000
## 14      +                          +   +            +                 8 -1012.207 2040.
4  65.01  0.000
## 22      +                          +   +                +             8 -1012.580 2041.
2  65.75  0.000
## 31      +          +               +   +            +   +            12 -1008.689 2041.
4  65.97  0.000
## 10      +                          +                +                 6 -1014.742 2041.
5  66.08  0.000
## 18      +                          +                    +             6 -1015.113 2042.
2  66.82  0.000
```

```
## 120      +                                       +    +         +    + 10 -1011.551 2043.
1  67.69  0.000
## 124      +                          +            +    +         +    + 12 -1009.571 2043.
1  67.74  0.000
## 69       +          +               +                                + 8 -1013.648 2043.
3  67.89  0.000
## 27       +          +          +                +    +                 10 -1011.772 2043.
5  68.14  0.000
## 30       +                      +    +           +    +                 10 -1012.205 2044.
4  69.00  0.000
## 68       +                          +                                + 6 -1016.330 2044.
7  69.25  0.000
## 26       +                      +                +    +                  8 -1014.741 2045.
5  70.08  0.000
## 65       +          +                                                + 6 -1016.753 2045.
5  70.10  0.000
## 64       +                                                           + 4 -1019.023 2046.
0  70.64  0.000
## 77       +          +               +            +                   + 10 -1013.493 2047.
0  71.58  0.000
## 85       +          +               +                 +              + 10 -1013.608 2047.
2  71.81  0.000
## 76       +                          +            +                   + 8 -1016.161 2048.
3  72.91  0.000
## 84       +                          +                 +              + 8 -1016.291 2048.
6  73.17  0.000
## 73       +          +                            +                   + 8 -1016.583 2049.
2  73.76  0.000
## 81       +          +                                 +              + 8 -1016.720 2049.
4  74.03  0.000
## 72       +                                       +                   + 6 -1018.842 2049.
7  74.28  0.000
## 80       +                                            +              + 6 -1018.992 2050.
0  74.58  0.000
## 93       +          +               +            +    +              + 12 -1013.454 2050.
9  75.50  0.000
## 92       +                          +            +    +              + 10 -1016.123 2052.
2  76.84  0.000
## 89       +          +                            +    +              + 10 -1016.553 2053.
1  77.70  0.000
## 88       +                                       +    +              + 8 -1018.812 2053.
6  78.22  0.000
## 37       +          +               +                      +             8 -1023.749 2063.
5  88.09  0.000
## 33       +          +                                      +             6 -1026.140 2064.
3  88.87  0.000
## 45       +          +               +            +         +            10 -1023.598 2067.
2  91.79  0.000
## 53       +          +               +                 +    +            10 -1023.649 2067.
3  91.89  0.000
## 41       +          +                            +         +             8 -1025.975 2067.
9  92.54  0.000
## 49       +          +                                 +    +             8 -1026.052 2068.
1  92.70  0.000
## 61       +          +               +            +    +    +            12 -1023.493 2071.
0  95.58  0.000
## 57       +          +                            +    +    +            10 -1025.884 2071.
8  96.36  0.000
```

```
## 36      +                                +                         +      6 -1032.298 2076.
6 101.19  0.000
## 32      +                                                          +      4 -1034.343 2076.
7 101.28  0.000
## 44      +                                +             +           +      8 -1032.175 2080.
4 104.94  0.000
## 52      +                                +                 +       +      8 -1032.193 2080.
4 104.98  0.000
## 40      +                                              +           +      6 -1034.210 2080.
4 105.01  0.000
## 48      +                                                  +       +      6 -1034.252 2080.
5 105.10  0.000
## 60      +                                +             +   +       +     10 -1032.069 2084.
1 108.73  0.000
## 56      +                                              +   +       +      8 -1034.118 2084.
2 108.83  0.000
## 5       +          +                    +                                6 -1040.351 2092.
7 117.30  0.000
## 13      +          +                    +             +                  8 -1040.223 2096.
4 121.04  0.000
## 1       +          +                                                     4 -1044.232 2096.
5 121.06  0.000
## 4       +                               +                                4 -1044.269 2096.
5 121.13  0.000
## 21      +          +                    +                 +              8 -1040.296 2096.
6 121.19  0.000
## 0       +                                                                2 -1047.528 2099.
1 123.65  0.000
## 9       +          +                                  +                  6 -1044.101 2100.
2 124.79  0.000
## 12      +                               +             +                  6 -1044.134 2100.
3 124.86  0.000
## 29      +          +                    +             +   +             10 -1040.173 2100.
3 124.94  0.000
## 17      +          +                                      +              6 -1044.185 2100.
4 124.96  0.000
## 20      +                               +                 +              6 -1044.205 2100.
4 125.00  0.000
## 8       +                                             +                  4 -1047.391 2102.
8 127.38  0.000
## 16      +                                                 +              4 -1047.475 2103.
0 127.54  0.000
## 25      +          +                                  +   +              8 -1044.059 2104.
1 128.71  0.000
## 28      +                               +             +   +              8 -1044.074 2104.
1 128.74  0.000
## 24      +                                             +   +              6 -1047.343 2106.
7 131.28  0.000
## Models ranked by AIC(x)
```

```
summary(model_dredge_avg)
```

```
##
## Call:
## model.avg(object = model_dredge, subset = delta <= 2)
##
```

```
## Component model call:
## multinom(formula = preference_modes_collapsed ~ <2 unique rhs>, data = acceptance_data,
weights = weight, na.action = na.fail)
##
## Component models:
##       df  logLik     AIC delta weight
## 1245  10 -977.70 1975.41  0.00   0.55
## 12345 12 -975.91 1975.83  0.42   0.45
##
## Term codes:
##                      Any_12M_dx efficacy_internet_intervention
 sex           treatment_experience                         intention
##                               1                              2
   3                              4                              5
##
## Model-averaged coefficients:
## (full average)
##                                            Estimate Std. Error z value Pr(>|z|)
## Digital((Intercept))                       -2.25790    0.18029  12.524  < 2e-16 ***
## Digital(Any_12M_dx1)                        0.43707    0.15863   2.755 0.005865 **
## Digital(efficacy_internet_interventionYes)  1.15610    0.16378   7.059  < 2e-16 ***
## Digital(treatment_experience1)             -0.78823    0.21297   3.701 0.000215 ***
## Digital(nointention/)                       1.01169    0.17547   5.766  < 2e-16 ***
## Blended((Intercept))                       -2.39934    0.20568  11.665  < 2e-16 ***
## Blended(Any_12M_dx1)                       -0.04013    0.22869   0.175 0.860703
## Blended(efficacy_internet_interventionYes)  1.01763    0.21880   4.651  3.3e-06 ***
## Blended(treatment_experience1)             -0.39374    0.26275   1.499 0.134000
## Blended(nointention)                        0.16548    0.20666   0.801 0.423285
## Digitalsexwoman)                           -0.09067    0.14133   0.642 0.521194
## Blended(sexwoman)                           0.09404    0.16978   0.554 0.579656
##
## (conditional average)
##                                            Estimate Std. Error z value Pr(>|z|)
## Digital((Intercept))                       -2.25790    0.18029  12.524  < 2e-16 ***
## Digital(Any_12M_dx1)                        0.43707    0.15863   2.755 0.005865 **
## Digital(efficacy_internet_interventionYes)  1.15610    0.16378   7.059  < 2e-16 ***
## Digital(treatment_experience1)             -0.78823    0.21297   3.701 0.000215 ***
## Digital(nointention)                        1.01169    0.17547   5.766  < 2e-16 ***
## Blended((Intercept))                       -2.39934    0.20568  11.665  < 2e-16 ***
## Blended(Any_12M_dx1)                       -0.04013    0.22869   0.175 0.860703
## Blended(efficacy_internet_interventionYes)  1.01763    0.21880   4.651  3.3e-06 ***
## Blended(treatment_experience1)              0.39374    0.26275   1.499 0.134000
-## Blended(non intention)                      0.16548    0.20666   0.801 0.423285
## Digital(sexwoman)                           0.20248    0.14822   1.366 0.171930
-## Blended(sexwoman)                           0.21000    0.20004   1.050 0.293810
## ---
## Signif. codes:  0 '***' 0.001 '**' 0.01 '*' 0.05 '.' 0.1 ' ' 1
```

```
model_final <- multinom(preference_modes_collapsed ~ Any_12M_dx + efficacy_internet_interve
ntion + sex + treatment_experience + intention, data = acceptance_data, weights = weight)
```

```
## # weights:  21 (12 variable)
## initial  value 1512.195310
## iter  10 value 1050.250812
## iter  20 value 975.920410
## final  value 975.913056
```

```
## converged
```

```
model_final %>% tbl_regression(estimate_fun = ~ style_number(.x, digits = 3)) %>% add_glanc
e_source_note()
```

```
## ℹ Multinomial models have a different underlying structure than the models
## gtsummary was designed for. Other gtsummary functions designed to work with
## tbl_regression objects may yield unexpected results.
```

Characteristic	log(OR)[1]	95% CI[1]	p-value
Digital			
Any_12M_dx			
0	—	—	
1	0.442	0.131, 0.753	0.005
perceived efficacy if IMIs			
No	—	—	
Yes	1.148	0.827, 1.470	<0.001
sex			
man	—	—	
woman	-0.202	-0.493, 0.088	0.2
treatment_experience			
0	—	—	
1	-0.768	-1.186, -0.351	<0.001
intention			
yes	—	—	
no/indifferent	1.011	0.667, 1.355	<0.001
Blended			

Any_12M_dx			
0	—	—	
1	-0.047	-0.495, 0.402	0.8
perceived efficacy of IMIs			
No	—	—	
Yes	1.026	0.597, 1.455	<0.001
sex			
man	—	—	
woman	0.210	-0.182, 0.602	0.3
treatment_experience			
0	—	—	
1	-0.414	-0.930, 0.103	0.12
intention			
yes	—	—	
no/indifferent	0.166	-0.239, 0.571	0.4

[1] OR = Odds Ratio, CI = Confidence Interval

edf = 12.0; Deviance = 1,952; AIC = 1,976; No. Obs. = 1,376

```
## ℹ Multinomial models have a different underlying structure than the models
## gtsummary was designed for. Other gtsummary functions designed to work with
## tbl_regression objects may yield unexpected results.
```

Characteristic	OR[1]	95% CI[1]	p-value
Digital			
Any_12M_dx			
0	—	—	
1	1.556	1.140,	0.005

		2.124	
perveiced efficacy of IMIs			
No	—	—	
Yes	3.153	2.287, 4.347	<0.001
sex			
man	—	—	
woman	0.817	0.611, 1.092	0.2
treatment_experience			
0	—	—	
1	0.464	0.306, 0.704	<0.001
intention			
yes	—	—	
no/indifferent	2.749	1.949, 3.878	<0.001
Blended			
Any_12M_dx			
0	—	—	
1	0.954	0.610, 1.494	0.8
percevied efficacy of IMIs			
No	—	—	
Yes	2.789	1.816, 4.284	<0.001
sex			
man	—	—	
		0.834,	

woman	1.234	1.826	0.3
treatment_experience			
0	—	—	
1	0.661	0.395, 1.108	0.12
intention			
yes	—	—	
no/indifferent	1.180	0.787, 1.769	0.4

[1] OR = Odds Ratio, CI = Confidence Interval

edf = 12.0; Deviance = 1,952; AIC = 1,976; No. Obs. = 1,376
